# Enhancing deep learning methods for brain metastasis detection through cross-technique annotations on SPACE MRI

**DOI:** 10.1186/s41747-025-00554-5

**Published:** 2025-02-06

**Authors:** Tassilo Wald, Benjamin Hamm, Julius C. Holzschuh, Rami El Shafie, Andreas Kudak, Balint Kovacs, Irada Pflüger, Bastian von Nettelbladt, Constantin Ulrich, Michael Anton Baumgartner, Philipp Vollmuth, Jürgen Debus, Klaus H. Maier-Hein, Thomas Welzel

**Affiliations:** 1 German Cancer Research Center (DKFZ) Heidelberg, Division of Medical Image Computing, Heidelberg, Germany; 2 Helmholtz Imaging, German Cancer Research Center (DKFZ), Heidelberg, Germany; 3 Faculty of Mathematics and Computer Science, Heidelberg University, Heidelberg, Germany; 4 Medical Faculty Heidelberg, University of Heidelberg, Heidelberg, Germany; 5 Division of Radiology, German Cancer Research Center (DKFZ), Heidelberg, Germany; 6 Department of Radiation Oncology, Heidelberg University Hospital, Heidelberg, Germany; 7 Department of Radiation Oncology, University Hospital Göttingen, Göttingen, Germany; 8 Heidelberg Institute of Radiation Oncology (HIRO), Heidelberg, Germany; 9 Clinical Cooperation Unit Radiation Oncology, German Cancer Research Center (DKFZ), Heidelberg, Germany; 10 Department of Neuroradiology, Heidelberg University Hospital, Heidelberg, Germany; 11 Division for Computational Neuroimaging, Heidelberg University Hospital, Heidelberg, Germany; 12 National Center for Tumor Diseases (NCT), NCT Heidelberg, a partnership between DKFZ and Heidelberg University Medical Center, Heidelberg, Germany; 13 German Cancer Consortium (DKTK), partner site Heidelberg, Heidelberg, Germany; 14 Heidelberg Ion-Beam Therapy Center (HIT), Department of Radiation Oncology, Heidelberg University Hospital, Heidelberg, Germany; 15 Division for Computational Radiology Clinical AI (CCIBonn.ai), Clinic for Neuroradiology, University Hospital Bonn, Bonn, Germany; 16 Medical Faculty Bonn, University of Bonn, Bonn, Germany; 17 Pattern Analysis and Learning Group, Radiation Oncology, Heidelberg University Hospital, Heidelberg, Germany; 18 Translational Lung Research Center Heidelberg (TLRC), Member of the German Center for Lung Research (DZL), Heidelberg, Germany

**Keywords:** Brain neoplasms, Deep learning, Image interpretation (computer-assisted), Image processing (computer-assisted), Magnetic resonance imaging

## Abstract

**Background:**

Gadolinium-enhanced “sampling perfection with application-optimized contrasts using different flip angle evolution” (SPACE) sequence allows better visualization of brain metastases (BMs) compared to “magnetization-prepared rapid acquisition gradient echo” (MPRAGE). We hypothesize that this better conspicuity leads to high-quality annotation (HAQ), enhancing deep learning (DL) algorithm detection of BMs on MPRAGE images.

**Methods:**

Retrospective contrast-enhanced (gadobutrol 0.1 mmol/kg) SPACE and MPRAGE data of 157 patients with BM were used, either annotated on MPRAGE resulting in normal annotation quality (NAQ) or on coregistered SPACE resulting in HAQ. Multiple DL methods were developed with NAQ or HAQ using either SPACE or MRPAGE images and evaluated on their detection performance using positive predictive value (PPV), sensitivity, and F1 score and on their delineation performance using volumetric Dice similarity coefficient, PPV, and sensitivity on one internal and four additional test datasets (660 patients).

**Results:**

The SPACE-HAQ model reached 0.978 PPV, 0.882 sensitivity, and 0.916 F1-score. The MPRAGE-HAQ reached 0.867, 0.839, and 0.840, the MPRAGE NAQ 0.964, 0.667, and 0.798, respectively (*p* ≥ 0.157). Relative to MPRAGE-NAQ, the MPRAGE-HAQ F1-score detection increased on all additional test datasets by 2.5–9.6 points (*p* < 0.016) and sensitivity improved on three datasets by 4.6–8.5 points (*p* < 0.001). Moreover, volumetric instance sensitivity improved by 3.6–7.6 points (*p* < 0.001).

**Conclusion:**

HAQ improves DL methods without specialized imaging during application time. HAQ alone achieves about 40% of the performance improvements seen with SPACE images as input, allowing for fast and accurate, fully automated detection of small (< 1 cm) BMs.

**Relevance statement:**

Training with higher-quality annotations, created using the SPACE sequence, improves the detection and delineation sensitivity of DL methods for the detection of brain metastases (BMs)on MPRAGE images. This MRI cross-technique transfer learning is a promising way to increase diagnostic performance.

**Key Points:**

- Delineating small BMs on SPACE MRI sequence results in higher quality annotations than on MPRAGE sequence due to enhanced conspicuity.
- Leveraging cross-technique ground truth annotations during training improved the accuracy of DL models in detecting and segmenting BMs.
- Cross-technique annotation may enhance DL models by integrating benefits from specialized, time-intensive MRI sequences while not relying on them.
- Further validation in prospective studies is needed.

**Graphical Abstract:**

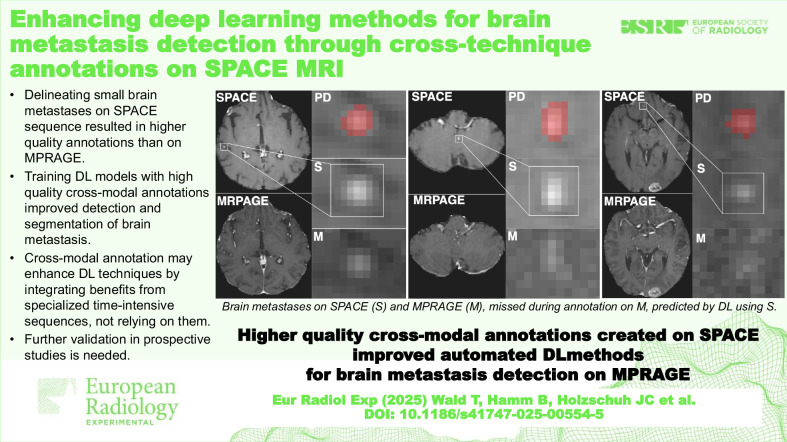

## Background

Approximately one in five patients with cancer will develop brain metastases (BMs) [1, 2]. A common therapy for BMs is stereotactic radiosurgery, which allows precise irradiation with excellent tumor control [3, 4]. Although stereotactic radiosurgery was historically recommended only for patients with up to four brain metastases [5], recent studies have demonstrated its successful application for up to ten brain metastases, regardless of their size [5, 6]. Therefore, highly sensitive detection of small BM is needed for optimal radiotherapy planning.

The typical magnetic resonance imaging (MRI) sequence for detecting BM is the late contrast-enhanced three-dimensional (3D) T1-weighted, commonly acquired through gradient-echo (GE) techniques such as magnetization-prepared rapid GE (MPRAGE) or similar sequences (*e.g*., BRAVO or 3D TFE) [7–9]. Recently, T1-weighted fast spin-echo black-blood (FSE-BB) sequences such as ‘sampling perfection with application-optimized contrasts using different flip angle evolution’ (SPACE) or similar sequences (*e.g*., CUBE or VISTA), gained prominence [10, 11]. These demonstrated higher contrast-to-noise ratio of BM relative to white matter [11–13], resulting in an improved overall sensitivity due to better conspicuity [11–16]. Moreover, black-blood-based imaging was shown to highly accurately predict treatment groups of patients with BMs [17].

Despite these advances, FSE-BB sequences have limitations in clinical practice; they cannot entirely replace GE sequences. For instance, vascular pathologies such as cerebral venous sinus thrombosis remain undetectable in these sequences due to the characteristic dark vasculature. Acquiring both GE and FSE-BB sequences can be time-consuming in routine clinical settings, posing practical challenges. Moreover, the SPACE sequence may not be applicable in all healthcare facilities, as older scanners do not support its acquisition and radiologists may not be trained to interpret SPACE images.

Given the complexity and critical nature of detecting BMs, numerous automated deep learning (DL) methods have been proposed. Some algorithms are designed for GE sequences [18–21], while others leverage the better conspicuity of BB-FSE sequences to maximize detection efficacy [22–26]. An overview was provided by Wang et al [27] and Ozkara et al [28]. While GE-only methods are widely applicable due to reliance on prevalent GE images, DL methods using BB-FSE images showed superior performance [24, 25] but require acquisition of an additional sequence.

In this study, we investigated if we can improve MPRAGE DL methods indirectly through higher annotation quality (HAQ) alone, created through delineation using the SPACE BB-FSE sequence. In doing so, BB-FSE images only have to be acquired during training dataset acquisition for cross-technique annotation generation, while the resulting DL model can be applied flexibly, saving potential time and cost of acquiring an additional MR image.

## Methods

### Image acquisition

For the development, training, and assessment of our models, data derived from the prospective CYBER-SPACE study [5, 29] was used. From April 2016 to February 2018, patients newly diagnosed with 1 to 10 BMs, irrespective of histological origin and size, were randomized in equal proportions to receive stereotactic radiosurgery for all identifiable lesions. Treatment was carried out in the Department of Radiation Oncology and Radiation Therapy at Heidelberg University Hospital, Germany. The decision on which lesions to target was based on imaging from one of two MRI sequences: either SPACE or MPRAGE, as defined in the original prospective study protocol. The pretherapeutic imaging was conducted within a particular timeframe at the local facility using a 3-T scanner (Magnetom Skyra®, Siemens Healthineers, Erlangen, Germany) and a quadrature four-channel head coil for transmit and receive purposes. Each scan was performed without the use of immobilization devices or axial tilting, allowing for the subsequent fusion with computed tomography scans in radiation treatment planning. In total, this study includes all 157 patients received. For details about cohort and primary tumor origin details, refer to Table 1.

**Table 1:** Dataset-wise patient details

	MPRAGE train set	SPACE train set	MPRAGE test set	SPACE test set	SUSM ataset*	UKHD dataset**	YNHH dataset	UMMC dataset
Patients, *n*	61	61	15	15	156 [105]	308	200	47
Females, *n* (%)	31 (50.8)	32 (52.5)	5 (33.3)	6 (40.0)	105 (67.3)	163 (52.9)	125 (62.5)	26 (55)
Males, *n* (%)	30 (49.2)	29 (47.5)	10 (66.7)	9 (60.0)	51 (32.7)	145 (47.1)	75 (37.5)	21 (45)
Age, median (IQR) or mean ± SD	63 (58–66)	64 (54–70)	61 (55–68)	61 (54–68)	Not available	61 ± 11	65 (55–73)	60 (53–66)
Primary tumor, *n* (%)								
Lung	39 (63.9)	41 (67.2)	11 (73.3)	11 (73.3)	99 (63.0)	124 (40.3)	103 (51.5)	22 (47.0)
Melanoma	12 (19.7)	10 (16.4)	2 (13.3)	3 (20.0)	7 (5.0)	18 (5.8)	41 (20.5)	2 (4.0)
Breast	4 (6.6)	4 (6.5)	2 (13.3)	1 (6.7)	33 (21.0)	68 (22.1)	26 (13.0)	11 (23.0)
Kidney	1 (1.6)	2 (3.3)	0 (0.0)	0 (0.0)	0 (0.0)	16 (5.2)	16 (8.0)	5 (11.0)
Unknown primary	0 (0.0)	2 (3.3)	0 (0.0)	0 (0.0)	0 (0.0)	18 (5.8)	0 (0.0)	0 (0.0)
Others	5 (8.2)	2 (3.3)	0 (0.0)	0 (0.0)	17 (11.0)	64 (20.8)	14 (7)	7 (15.0)
Previous brain radiotherapy, *n* (%)								
No SRS/WBRT	61 (100.0)	60 (98.4)	15 (100.0)	15 (100.0)	156 (100.0)	308 (100.0)	200 (100.0)	47 (100.0)
SRS	0 (0.0)	1 (1.6)	0 (0.0)	0 (0.0)	0 (0.0)	0 (0.0)	0 (0.0)	0 (0.0)
WBRT	0 (0.0)	0 (0)	0 (0.0)	0 (0)	0 (0.0)	0 (0.0)	0 (0.0)	0 (0.0)
Lesion-wise volumes (cm^3^)								
Median (IQR)	0.13 (0.04–0.48)	0.11 (0.03–0.58)	0.15 (0.04–0.51)	0.26 (0.045–1.35)	0.04 (0.02–0.14)	0.05 (0.01–0.29)	0.12 (0.04–0.69)	0.24 (0.06–1.15)
Mean ± SD	0.83 ± 2.74	0.89 ± 2.23	0.62 ± 1.11	1.29 ± 2.40	0.42 ± 2.52	1.15 ± 4.43	1.5 ± 4.32	2.75 ± 7.46

Patient details for all internal and additional test cohorts used in this study. Of all studies, we only used the contrast-enhanced T1-weighted gradient-echo sequences

*IQR* Interquartile range, *SD* Standard deviation, *SRS* Stereotactic surgery, *SUSM* Stanford University School of Medicine, *UKHD* University Clinic Heidelberg, *UMMC* University of Mississippi Medical Center, *WBRT* Whole-breast radiation therapy, *YNHH* Yale New Heaven Hospital

* For the SUSM dataset. Only 105 patients with annotations are publicly available, yet patient details are not provided, hence we report the patient information from the original paper of all 156 patients, with the lesion volume being the exception as we could calculate this ourselves

** The UKHD dataset was comprised of all training and test cases of the original study, as we did not train on the additional test datasets

### Imaging protocol

For each patient, four MRIs and one computed tomography scan were acquired: unenhanced MPRAGE, contrast-enhanced early-phase MPRAGE, contrast-enhanced late-phase MPRAGE, and a contrast-enhanced late-phase SPACE sequence. A uniform imaging protocol was employed for all patients. The MPRAGE sequence was 3D high-resolution T1-weighted (repetition time 2000 ms, echo time 2.44 ms, slice thickness 0.9 mm), acquired before and after 2 and 10 min after contrast injection. Contrast agent was administered via intravenous injection of 0.1 mmol/kg body weight gadobutrol (Gadovist, Bayer AG, Berlin, Germany) and a supplementary 30 mL of saline flush. The SPACE sequence parameters included a repetition time of 700 ms, an echo time of 12 ms, a flip angle of 120°, and a slice thickness of 1.0 mm. There were no complementary high-resolution T2-weighted images acquired as acquisition times would have been too long for patients to tolerate.

### Image preprocessing

MRI images were affinely coregistered to CT images for later radiation therapy by a radiologist (T.We.) and radiation oncologist (S.R,), both with over 15 years of cerebral MRI expertise. They reviewed the MRI acquisitions and created ground truth (GT) segmentations of BMs using the ‘Multiplan’ treatment planning system (Accuray Inc., Sunnyvale, US). For the first cohort, GTs were created on the late-phase contrast-enhanced MPRAGE images, while in the second cohort, they were created on the contrast-enhanced SPACE images. All identified lesions were included, irrespective of size. For the development of the DL methods, all images were brain extracted using the fully automatic DL brain extraction tool HD-BET described by Isensee et al [30].

### Datasets

#### Internal dataset

**Fig. 1:**
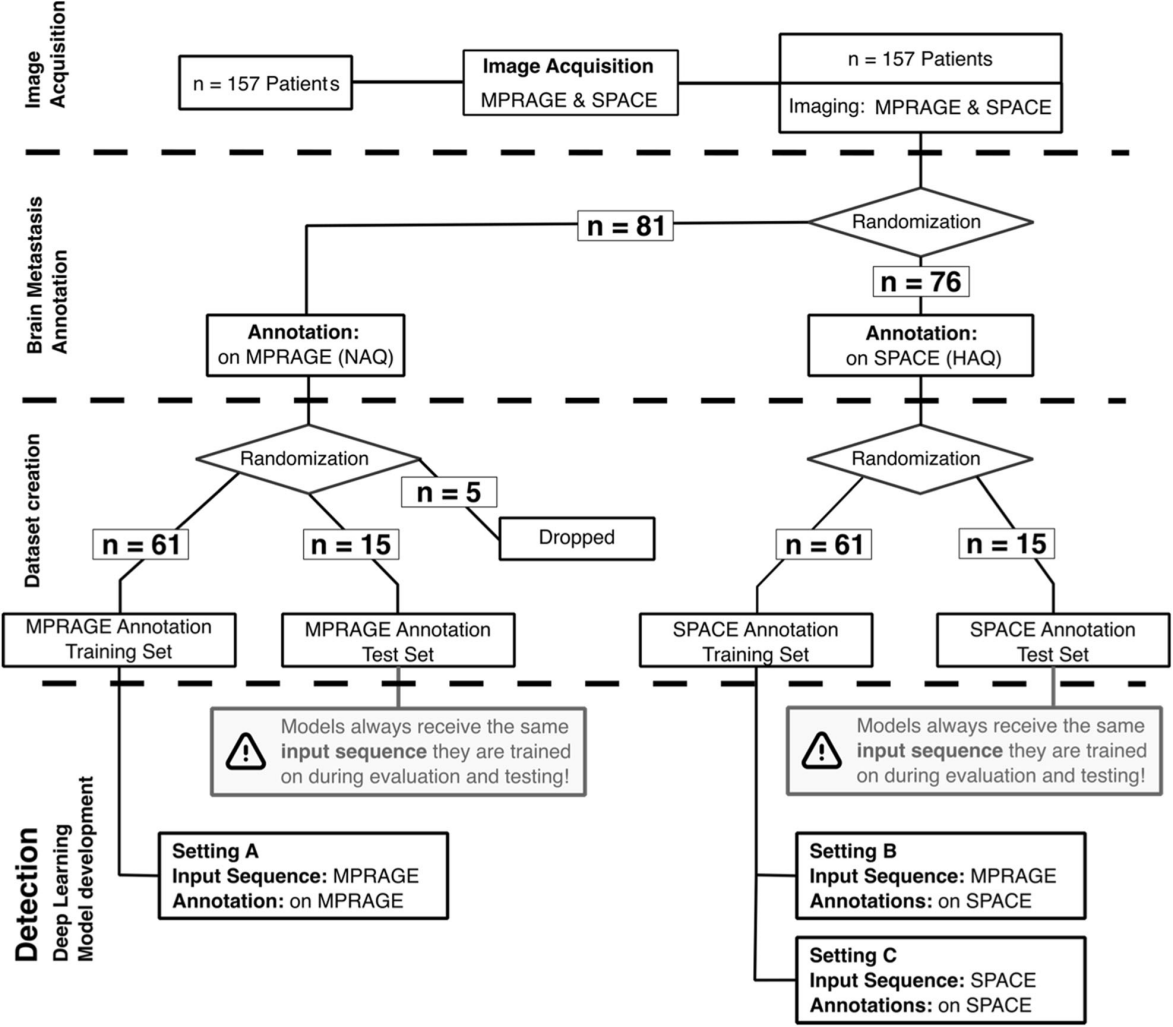
Outline of the selection and preparation process of patient data for deep learning method development utilizing patients from the CYBER-SPACE study. A total of 157 patients were provided, with imaging performed using MPRAGE and SPACE modalities. In the original study, patients were randomized into two cohorts to delineate BMs either on MPRAGE or SPACE for later radiation treatment. Of the original study, 76 patients delineated on SPACE and 81 delineated on MPRAGE were received of which 5 were dropped randomly for equal cohort sizes. Due to lower conspicuity of BM on MPRAGE annotations are of normal annotation quality (NAQ), whereas annotations created on SPACE result in high annotation quality (HAQ). Given these images and annotations models were trained in three settings: A—MPRAGE image as input with NAQ; B—MPRAGE image as input with HAQ; and C—SPACE images and HAQ. The flowchart details the distribution of patients at each differentiation step of the study. MPRAGE, Magnetization-prepared rapid acquisition gradient echo; SPACE, Sampling perfection with application-optimized contrasts using different flip angle evolution

Of the 157 patients, 81 had BMs delineated on MPRAGE and 76 on SPACE. Several anatomical structures were annotated, most notably the planning target volume, gross tumor volume, and clinical target volume for all brain metastases, using the “CyberKnife center” (Accuray Inc.). In the larger MPRAGE cohort, 5 patients were randomly excluded to equalize the cohort sizes. These cohorts were split into train/validation and test datasets in a 4:1 ratio, resulting in two train/validation datasets, each with 61 patients, and two test datasets, each with 15 patients. In the following, what was initially referred to as the “train/validation dataset” will be called simply the “train set”. For dataset creation details, refer to Fig. 1, and for patient and BMs details, refer to Table 1. While both SPACE and MPRAGE images were available for all patients, annotations were initially created for only one of the two sequences during the prospective trial. The retrospective manual addition of annotations to the other sequence was not conducted due to the associated high costs. Furthermore, semiautomatic annotation was avoided to prevent the possibility of influencing the expert annotators’ behavior through biases introduced by the supporting deep learning model.

#### Additional test datasets

Four additional BMs test datasets comprising 660 additional patients were used to test the models. Specifically, the publicly available Stanford University School of Medicine (SUSM) BrainMetShare dataset [22] hosted at https://aimi.stanford.edu/brainmetshare, the publicly available Yale New Heaven Hospital (YNHH) Pretreat-MetsToBrain-Masks dataset [31, 32], and the University of Mississippi Medical Center dataset (UMMC) GammaKnife dataset [31, 33], both accessible through The Cancer Imaging Archive—TCIA (https://www.cancerimagingarchive.net/), as well as the proprietary dataset from the University Clinic Heidelberg (UKHD) [21]. For dataset details, refer to Table 1.

### Experimental design

#### Settings definition

For all patients both MPRAGE and SPACE images were acquired, even if not used for BM delineation. To assess the importance of image and annotation quality we trained DL models in three different settings:

- A—the model is trained with the contrast-enhanced MPRAGE images and annotations derived from the very same MPRAGE images (MPRAGE annotation group, NAQ);
- B—the model is trained with the contrast-enhanced MPRAGE images and annotations derived from co-registred SPACE images (SPACE annotation group, HAQ);
- C—the model is trained with the contrast-enhanced SPACE images and annotations derived from the very same SPACE images (SPACE annotation group, HAQ).

Of these, setting A represents the current default for all DL methods that are trained without any black-blood imaging [18–21], setting B represents the cross-technique annotation setting with the model leveraging HAQ created with the help of the SPACE sequence but still using MPRAGE images as input for the DL method, and setting C represents a model that uses both, SPACE (or other BB-FSE) images as input and HAQ [22–26]. There exists another setting using SPACE images with coregistered NAQ annotations, which we refer to as setting D. Due to it not being clinically relevant, we provide its evaluation in the Supplemental material.

#### Deep learning model development

The convolutional neural network (CNN) architectures we employed are based on the self-configuring nnU-Net method [34], a state-of-the-art framework that has shown tremendous performance and won multiple challenges [35, 36]. For each setting, we trained a 5-fold cross-validated model for the respective training dataset with an 80:20 split and evaluated the ensemble on the test datasets. Preprocessing, hyperparameters, and training details were automatically configured according to the nnU-Net v1 framework and ‘nnUNetTrainerV2’. For further details, we refer to the repository https://github.com/MIC-DKFZ/nnUNet/.

### Evaluation metrics and statistical analysis

Prediction and GT instances were created through connected component analysis and subsequently matched based on their Dice similarity coefficient (DSC). Instances were considered detected when the DSC between the instance and its matched prediction exceeded 0.1. Based on this matching detection performance is measured through positive predictive value (PPV), sensitivity, and F1 score. Specificity and negative predictive value cannot be reported, as no true negative could be calculated. Segmentation performance was evaluated by the per-patient lesion-wise volumetric DSC, PPV, and sensitivity. As different patients have different numbers of BMs, we aggregate these values per-patient before aggregating across the dataset. To test statistical significance, we employed two-sided Mann–Whitney *U* tests due to normality constraints. More details on the evaluation metrics and statistical analysis are provided in Appendix A.

## Results

For brevity, ‘the model trained in setting A’ is referred to as ‘model A’. Values for mean ± standard deviation are reported for the respective models in alphabetical order. More detailed metrics and all associated *p*-values are provided in Tables 2 and 3.

**Table 2:** Comprehensive detection and delineation performance metrics of model A and model B on internal test datasets

		Model A		Model B		
Dataset	Metric	Mean ± SD	Median (IQR)	Mean ± SD	Median (IQR)	*p*-value
SPACE	Instance F1 score	74.5 ± 27.8	80.0 (60.6–100.0)	84.0 ± 17.6	88.9 (66.7–100.0)	2.36E-01
	Instance PPV	96.7 ± 12.9	100.0 (100.0–100.0)	86.7 ± 19.1	100.0 (75.0–100.0)	1.72E-01
	Instance sensitivity (%)	66.7 ± 30.4	66.7 (50.0–100.0)	83.9 ± 19.7	100.0 (66.7–100.0)	2.77E-02
	LW voxel DSC (%)	60.7 ± 24.4	64.7 (47.0–81.0)	64.4 ± 14.0	60.6 (54.5–75.9)	8.04E-01
	LW voxel PPV (%)	68.1 ± 25.3	72.5 (58.3–85.7)	72.5 ± 19.8	79.5 (54.6–90.7)	8.47E-01
	LW voxel sensitivity (%)	59.0 ± 25.1	61.6 (43.5–75.1)	61.2 ± 13.2	61.1 (50.6–69.6)	5.61E-01
MPRAGE	Instance F1 Score	91.9 ± 11.2	100.0 (85.7–100.0)	89.5 ± 14.9	100.0 (85.7–100.0)	2.85E-01
	Instance PPV	97.8 ± 8.6	100.0 (100.0–100.0)	91.1 ± 18.8	100.0 (100.0–100.0)	1.57E-01
	Instance sensitivity	89.0 ± 17.2	100.0 (75.0–100.0)	90.3 ± 15.5	100.0 (77.5–100.0)	3.17E-01
	LW voxel DSC	68.3 ± 18.4	70.2 (58.5–78.2)	68.8 ± 14.6	66.0 (59.3–78.7)	3.03E-01
	LW voxel PPV	74.1 ± 14.9	72.4 (61.9–85.7)	70.2 ± 16.5	70.2 (59.6–83.9)	1.51E-01
	LW voxel sensitivity	70.1 ± 21.0	72.8 (59.7–84.0)	71.9 ± 19.0	72.9 (63.1–86.1)	3.30E-01

Data are mean percentages ± SD or median percentages (IQR, first–third quartile) and *p*-values between the models for each of the metrics

*DSC* Dice similarity coefficient, *IQR* Interquartile range, *LW* Lesion-wise, *MPRAGE* Magnetization-prepared rapid acquisition gradient echo, *PPV* Positive predictive value, *SD* Standard deviation, *SPACE* Sampling perfection with application-optimized contrasts using different flip angle evolution

**Table 3:** Comprehensive detection and delineation performance metrics of models A and B on the four additional test datasets

	Metric	Model A		Model B		
		Mean ± SD	Median (IQR)	Mean ± SD	Median (IQR)	*p*-value
SUSM	Instance F1 score	59.6 ± 29.7	66.7 (44.4–80.0)	68.2 ± 24.4	69.2 (60.0–84.6)	8.81E-05
	Instance PPV	83.0 ± 27.7	100.0 (75.0–100.0)	81.2 ± 25.7	95.8 (66.7–100.0)	4.34E-01
	Instance sensitivity	53.1 ± 31.1	50.0 (33.3–75.0)	64.8 ± 28.4	66.7 (50.0–100.0)	1.00E-08
	LW voxel DSC	40.1 ± 22.6	40.8 (27.7–54.2)	46.7 ± 20.8	49.1 (34.8–61.4)	4.66E-06
	LW voxel PPV	51.6 ± 24.8	54.5 (39.0–67.4)	55.4 ± 20.3	57.9 (44.6–66.6)	4.51E-02
	LW voxel sensitivity	39.1 ± 24.6	37.1 (25.6–53.0)	46.7 ± 22.0	48.1 (30.8–63.2)	1.28E-06
YNHH	Instance F1 score	70.3 ± 31.2	72.7 (52.5–100.0)	75.2 ± 27.9	81.7 (66.7–100.0)	6.20E-03
	Instance PPV	90.9 ± 20.6	100.0 (100.0–100.0)	84.5 ± 27.0	100.0 (75.0–100.0)	5.26E-04
	Instance sensitivity	66.6 ± 34.1	72.6 (42.9–100.0)	75.1 ± 29.5	84.5 (50.0–100.0)	8.75E-08
	LW voxel DSC	48.1 ± 25.7	49.4 (32.1–70.1)	52.0 ± 23.1	54.8 (36.9–70.7)	8.21E-04
	LW voxel PPV	47.5 ± 22.7	51.6 (33.4–63.8)	51.9 ± 20.7	55.1 (38.7–65.1)	1.14E-01
	LW voxel sensitivity	56.6 ± 32.3	59.1 (35.0–86.4)	61.5 ± 29.6	64.7 (41.0–88.3)	1.12E-05
UMMC	Instance F1 score	64.6 ± 31.9	66.7 (63.8–85.7)	74.2 ± 31.4	80.0 (66.7–100.0)	7.51E-04
	Instance PPV	74.5 ± 31.9	83.3 (50.0–100.0)	86.1 ± 26.9	100.0 (81.7–100.0)	4.16E-03
	Instance sensitivity	69.0 ± 35.9	75.0 (50.0–100.0)	75.3 ± 33.0	100.0 (50.0–100.0)	5.81E-02
	LW voxel DSC	47.3 ± 26.6	52.4 (31.0–61.2)	55.8 ± 28.4	60.0 (40.0–76.3)	1.29E-04
	LW voxel PPV	60.6 ± 29.7	65.0 (57.0–79.4)	70.4 ± 28.2	74.5 (62.7–93.7)	5.40E-03
	LW voxel sensitivity	44.0 ± 28.9	44.1 (21.3–62.3)	51.5 ± 29.4	56.4 (29.9–76.7)	1.02E-03
UKHD	Instance F1 score	72.7 ± 29.1	80.0 (59.8–100.0)	75.2 ± 26.0	80.0 (64.2–100.0)	1.60E-02
	Instance PPV	80.0 ± 28.4	100.0 (66.7–100.0)	79.0 ± 26.4	92.0 (64.1–100.0)	3.33E-01
	Instance sensitivity	74.4 ± 31.2	91.3 (50.0–100.0)	79.0 ± 27.6	100.0 (58.8–100.0)	4.74E-07
	LW voxel DSC	47.8 ± 24.7	49.9 (31.8–66.4)	48.8 ± 22.6	50.5 (33.5–65.4)	5.63E-01
	LW voxel PPV	43.9 ± 22.3	45.0 (29.3–58.3)	43.5 ± 20.5	43.9 (29.3–58.3)	2.08E-01
	LW voxel sensitivity	64.7 ± 29.6	66.6 (47.8–92.1)	68.3 ± 27.6	73.1 (50.9–94.9)	3.05E-04

Data are mean percentages ± SD or median percentages (IQR, first–third quartile) and *p*-values between the models for each of the metrics

*DSC* Dice similarity coefficient, *IQR* Interquartile range, *LW* Lesion-wise, *PPV* Positive predictive value, *SD* Standard deviation

### Detection performance

**Fig. 2:**
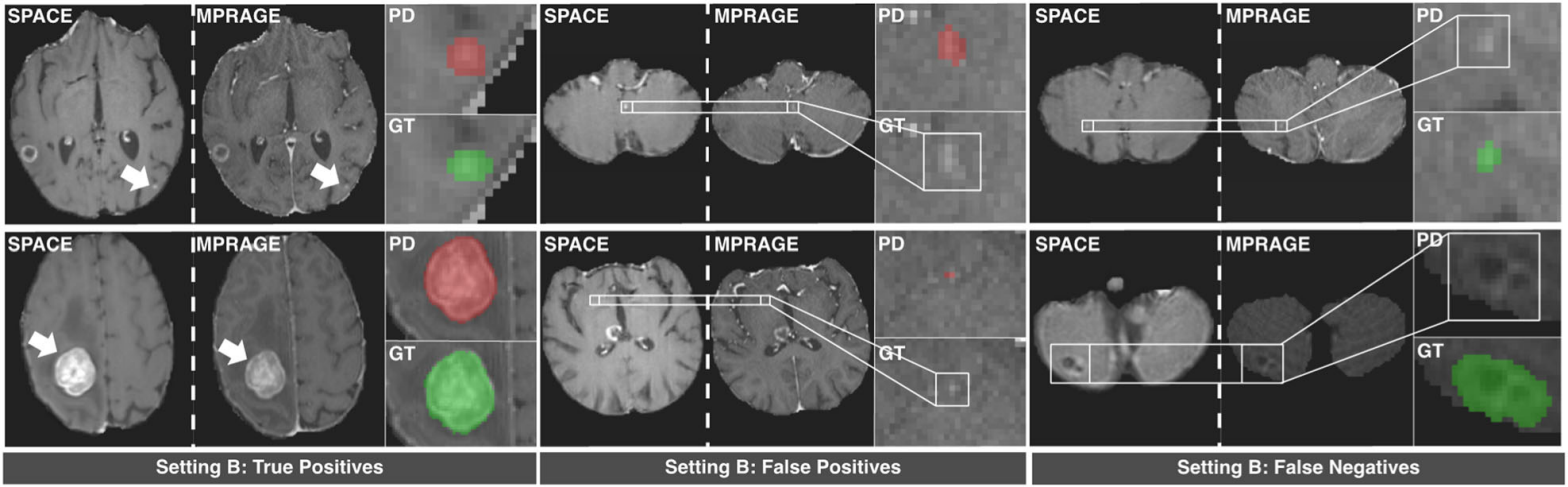
Results of the model trained in setting B on the SPACE test set. We display true positive, false positive, and false negative instances in comparison to the ground truth (GT). GT is marked in green, while the model predictions (PD) are colored red. Images display axial contrast-enhanced SPACE and contrast-enhanced MPRAGE images as acquired during the CYBER-SPACE study. MPRAGE, Magnetization-prepared rapid acquisition gradient echo; SPACE, Sampling perfection with application-optimized contrasts using different flip angle evolution

Results on the internal test datasets are reported in order of model A, model B, model C. On the HAQ SPACE test set the models achieved a F1 Score of 0.798 ± 0.193, 0.840 ± 0.175, 0.916 ± 0.139, a PPV of 0.964 ± 0.134, 0.867 ± 0.191, 0.978 ± 0.086, and a sensitivity of 0.667 ± 0.304, 0.839 ± 0.197, 0.881 ± 0.193 (Table 4). Qualitative visualization of predictions of model B is provided in Fig. 2.

**Table 4:** Internal detection performance metrics of models A, B, and C on the internal SPACE and MPRAGE test sets

Dataset	MPRAGE test set			SPACE test set		
Metrics	Instance F1 score	Instance PPV	Instance sensitivity	Instance F1 score	Instance PPV	Instance sensitivity
Model A	91.9% ± 11.2%	97.8% ± 8.6%	89.0% ± 17.2%	79.8% ± 19.4%	96.4% ± 13.4%	66.7% ± 30.4%
Model B	89.5% ± 14.9%	91.1% ± 18.8%	90.3% ± 15.5%	84.0% ± 17.6%	86.7% ± 19.1%	83.9% ± 19.7%
Model C	87.8% ± 14.6%	84.7% ± 19.5%	94.0% ± 12.8%	91.6% ± 13.9%	97.8% ± 8.6%	88.1% ± 19.3%

Data are means ± SD for F1 score, PPV, and sensitivity of each model on each test dataset

*PPV* Positive predictive value, *SD* Standard deviation, *MPRAGE* Magnetization-prepared rapid acquisition gradient echo, *SPACE* Sampling perfection with application-optimized contrasts using different flip angle evolution

Quantifying the model performance on the HAQ SPACE test set, model A shows the lowest F1 score, driven by low sensitivity despite having a high PPV. Model B, predicting the same images, had +4.2 points of F1 score, -9.7 points of PPV but +17.2 points increase in sensitivity, relative to model A. Model C, the upper baseline using SPACE images, reached +11.8 points of F1 score, +0.1 points of PPV, and +21.3 points of sensitivity, relative to model A.

Although pairwise comparisons did not show statistical significance (*p* ≥ 0.171), Model B demonstrated a clear performance improvement over model A in terms of F1 score and sensitivity, closing 40% of the gap to model C. Notably, both models received identical MPRAGE input images, differing only in the annotations used during training (model A MPRAGE, NAQ; model B MPRAGE, HAQ).

On the additional test datasets, only model A and model B are applicable due to lack of FSE-BB imaging. To better visualize the change in model performance we report the difference (B minus A) and the *p*-value, with positive values indicating a better performance of the model trained with HAQ cross-modal annotations.

**Fig. 3:**
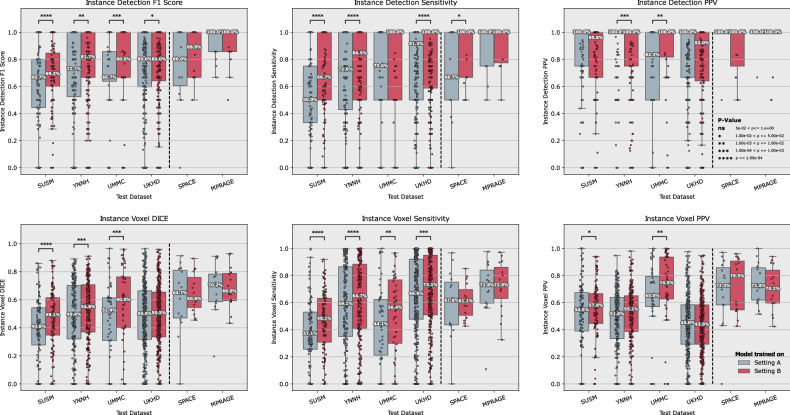
Comparative performance of the model trained in setting A (gray) and B (red) across multiple datasets for various diagnostic metrics. The top row represents per-patient lesion-wise detection metrics of F1 score, sensitivity, and PPV. Similarly, the bottom row represents the per-patient lesion-wise volumetric voxel DSC, voxel sensitivity, and voxel PPV. The model developed in setting B demonstrates superior sensitivity across several datasets, as indicated by the higher median values and more favorable interquartile ranges in the corresponding plots. This comprehensive overview highlights the enhanced ability of setting B to accurately identify and measure brain metastases, despite predicting on the same MPRAGE image. The effect is particularly strong in terms of sensitivity, which is critical for reliable detection, assessment and Stereotactic Radiosurgery. Corresponding means ± standard deviations, median (interquartile range), and *p*-values can be found in Tables 3 and 4. DSC, Dice similarity score; PPV, Positive predictive value, MPRAGE, Magnetization-prepared rapid acquisition gradient echo; SPACE, Sampling perfection with application-optimized contrasts using different flip angle evolution

Regarding F1 score, model B demonstrated clearly superior performance compared to model A across all additional test datasets, achieving a statistically significant improvement of +8.6 (*p* < 0.001), +4.9 (*p* < 0.006), +9.6 (*p* < 0.001), and +2.5 (*p* = 0.016) on the SUSM, YNHH, UMMC, and UKHD datasets, respectively. While PPV did not increase in all cases, a statistically significant increase of +11.6 (*p* < 0.004) was shown for the UMMC dataset, while PPV decreased by -1.8 (*p* = 0.434), -6.4 (*p* < 0.001), and -1.0 (*p* = 0.333) for the SUSM, YNHH, and UKHD dataset, respectively. Notably, the significance level was reached only for YNHH. For sensitivity, model B also performed significantly better across all datasets with a sensitivity of +11.7 (*p* < 0.001), +8.4 (*p* < 0.001), +6.3 (*p* = 0.058), and +4.6 (*p* < 0.001) for the SUSM, YNHH, UMMC, and UKHD, respectively. A comparative visualization and detailed values are shown in Fig. 3 and Tables 3 and 4.

For all datasets, model B reached statistically significantly higher F1 Scores than model A and for 3 out of 4 datasets it exhibits statistically significant improvements in Sensitivity over model A.

**Fig. 4:**
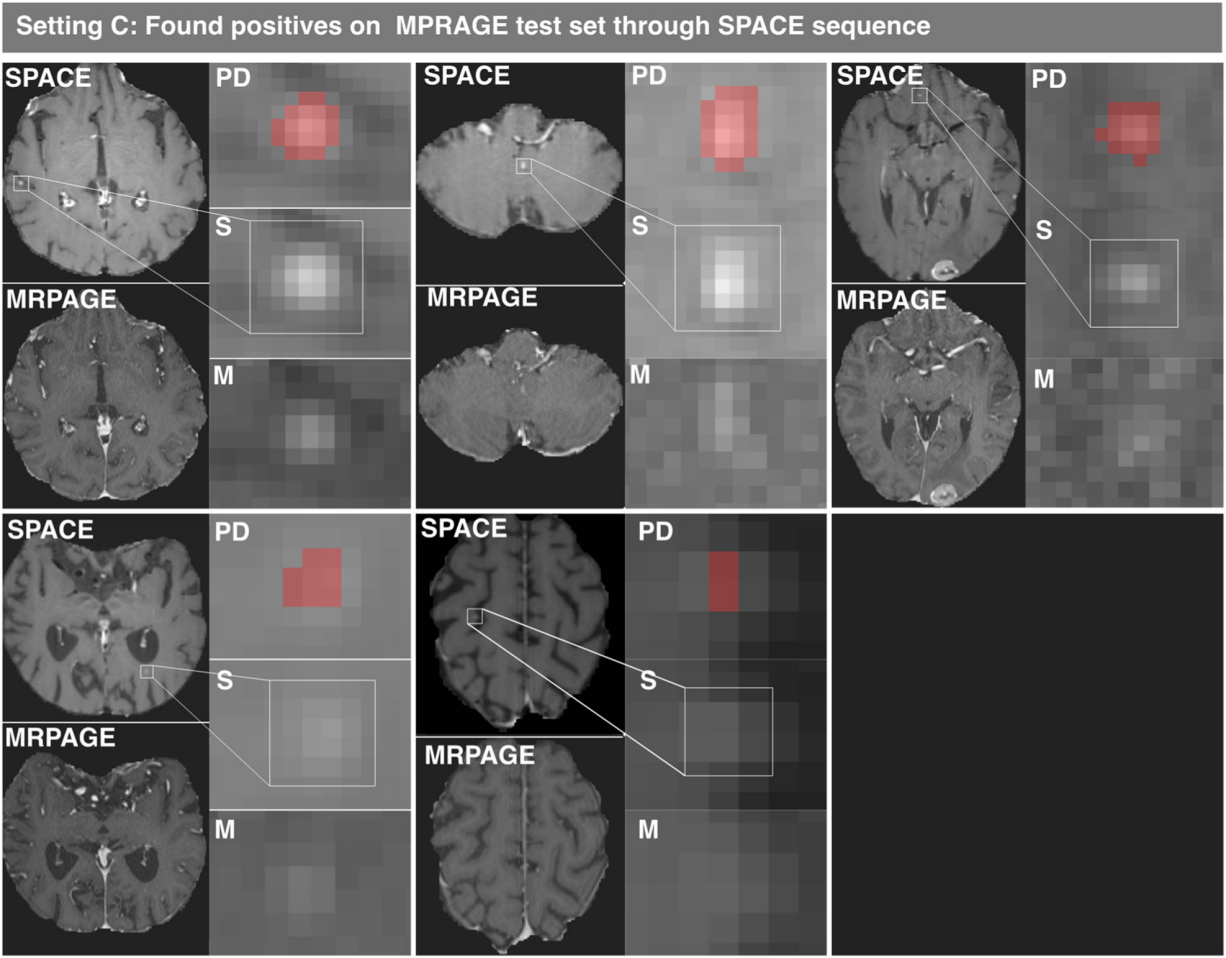
Qualitative visualization of supposed false positive predictions of model C on the MPRAGE test set, where five false positives were initially reported. Subsequent evaluation using SPACE revealed these false positives to be true positives, which had been missed by experts on the MPRAGE images alone. The red voxels highlight the regions where the model predicted (PD), and no GT was annotated, but which was later confirmed as being metastases. S shows the region of interest on axial contrast-enhanced SPACE and M on axial contrast-enhanced MPRAGE respectively. MPRAGE, Magnetization-prepared rapid acquisition gradient echo; SPACE, Sampling perfection with application-optimized contrasts using different flip angle evolution

Curiously, PPV of model C decreased substantially on the internal NAQ MPRAGE test set from 0.978 ± 0.086 to 0.847 ± 0.195 (Table 2). Consequently, the false positive predictions were investigated by the expert radiologist T.We. with the help of the SPACE sequence. Of 7 false positives predicted by model C, 5 predicted instances were found to be BMs missed during annotation on only MPRAGE images. The remaining two false positives were an aneurysm and a vessel. The found instances are visualized in Fig. 4. A similar analysis for model B showed 2 out of 3 total false positives to be correct predictions of the same BM instances. Note that model B, unlike model C, predicted on the same images that were used by the radiologist for annotation. Model A, trained with NAQ and MPRAGE images found none of these missed instances.

We do not report adjusted F1 scores, PPV, and sensitivity for these missed instances as this would introduce a bias that would unfairly advantage the models that were used to find these instances. More specifically, there are likely additional instances that the models did not find that are visible on SPACE which an annotator would have found, that would not be taken into account. This would inflate the perceived performance of the HAQ models. Hence, to accurately calculate new F1 scores, PPV, and sensitivity, it would be necessary to re-examine all images and assess for false negatives on these as well.

### Segmentation performance

For internal datasets models A, B, and C achieved a volumetric DSC of 0.607 ± 0.244, 0.644 ± 0.140 and 0.722 ± 0.140, a PPV of 0.681 ± 0.253, 0.725 ± 0.198, and 0.750 ± 0.138, and a sensitivity of 0.590 ± 0.251, 0.612 ± 0.132 and 0.727 ± 0.177, respectively, with none of them statistically significant (*p* ≥ 0.561).

For the additional test datasets, we report results as difference (B minus A) and *p*-value, analog as in the detection setting above.

Across all additional test datasets, DSC improvements were observed with +6.6 (*p* < 0.001) for the SUSM dataset, +3.9 (*p* < 0.001) for the YNHH dataset, +8.5 (*p* < 0.001) for the UMMC dataset, and +1.0 (*p* = 0.563) for the UKHD. PPV showed a variable performance with increases of +3.8 (*p* = 0.045) for the SUSM dataset, +4.4 (*p* = 0.114) for the YNHH dataset, +10.2 (*p* = 0.005) for the UMMC dataset, and a slight decrease of -0.4 (*p* = 0.208) for the UKHD dataset. Sensitivity also demonstrated improvements with +7.6 for the SUSM dataset, +4.9 for the YNHH dataset, +7.5 for the UMMC dataset, and +3.6 for the UKHD dataset (*p* < 0.001 for all).

For three out of the four additional test datasets, overall lesion-wise DSC segmentation performance improves statistically significantly when the model was developed with HAQ as opposed to NAQ. Similarly, on all additional test datasets, volumetric sensitivity of model B is significantly higher relative to model A, indicating more tumor volume being delineated.

### Overall found instances

**Fig. 5:**
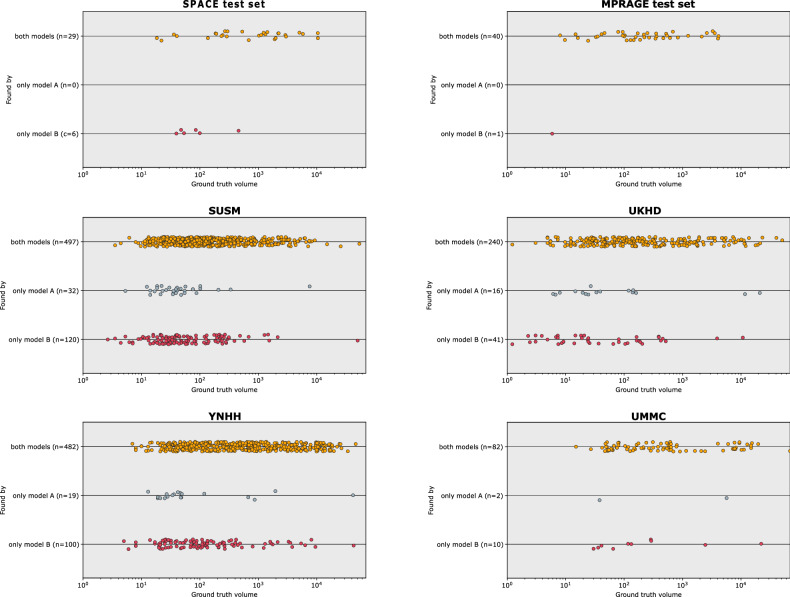
Comparison of detection of brain metastases across different datasets using models A and B. Each plot represents one dataset and illustrates which BMs were found by both models A and B (yellow), found by model A only (gray) or found by model B only (red). The position of the points on the *x*-axis indicates instance volume with instances on the left being smaller and instances on the right being larger. The plots collectively emphasize the enhanced detection capabilities of model B over model A, as evident by the greater number of red points across datasets. It also indicates the superiority of model B over model A for smaller brain metastases and overall higher difficulty in detecting small lesions

Comparing instance detection between model A and model B across all datasets, ordered as SUSM, YNHH, UMMC, UKHD, SPACE, and MPRAGE, Model B detected 120, 100, 10, 41, 6, and 1 instances missed by model A, while model A found 32, 19, 2, 16, 0, and 0 instances overlooked by model B. This corresponds to model B finding about 0.84, 0.405, 0.17, 0.08, 0.4, and 0.07 additional BM instances per patient relative to model A (Fig. 5).

## Discussion

In this study we investigated whether DL methods can benefit from HAQ created with the help of the SPACE sequence without actually using the SPACE images as input to the DL model. Overall, our results demonstrated that model B consistently outperformed model A across a range of internal and additional test datasets on multiple metrics. Notably, both models processed identical input images, differing solely in their training methodologies as model B was trained using SPACE annotations (HAQ), whereas model A utilized MPRAGE annotations (NAQ). Thus, we deem this a potent way to improve the performance of DL methods applied to contrast-enhanced MPRAGE sequences for the detection and delineation of BMs without requiring FSE-BB images at test time, making it widely applicable. While performance increased substantially, the model could not compensate fully for the lack of better conspicuity provided by FSE-BB images, which is in line with existing literature [24].

Moreover, our study highlights that MPRAGE sequences do not always provide a fully sufficient basis to highly sensitively detect and delineate BM lesions. In our internal test set (*n* = 15), where contrast-enhanced MPRAGE was used for delineation, we identified 5 previously missed BMs after reviewing the supposed false positive predictions of our DL model C. Not only does this lower annotation quality negatively impact DL model performance, but it also implicates that in clinical practice, BMs may be missed when relying solely on GE sequences, potentially leading to suboptimal decisions in stereotactic radiosurgery therapy.

Aside from actual model performance, our results suggest that annotation quality strongly influences measured model performance. Reported performance of existing DL methods [18–28] on datasets that do not leverage FSE-BB sequences for GT annotation may provide overconfident performance estimates [11–16]. Additionally, a direct comparison of published methods on internal proprietary datasets [27, 28] is less reliable as performance is highly influenced by the annotation quality. Thus, we advocate for comparisons on publicly available datasets [22, 32, 33] if models aim for maximal performance and comparable data is available.

In a broader context of DL research in MRI, the concept of utilizing complementary information across various image sequences or modalities to refine GT shows great potential and can be extended beyond the specific application shown in this study, also to imaging modalities other than MRI. Annotations created on a suboptimal modality constrain the model to learn only what the expert has seen on this specific modality. This inherently inhibits models trained using supervised learning from reaching superhuman performance due to the limited accuracy of the examples provided. However, cross-technique or cross-modality annotations may enable a model to discern subtle image features that are invisible to the human eye (as happened in this study with a model created on an MRI sequence with improved conspicuity of the pathology).

Our study has limitations. First, we acknowledge the relatively small single-center training and test dataset in our internal settings. A larger training and test set would increase the overall performance of the models and would allow more statistically significant conclusions on the internal sets. This noted, it is to emphasize that this study is not aimed at building the best possible model for application in a clinical setting. Instead, the benefit of HAQ relative to the NAQ was investigated—with both models built in an equally constrained setting—hence, in this conceptual framework, absolute performance was secondary.

To achieve reliable conclusions, we evaluated both methods on a wide range of additional test datasets, which reduced DL model performance further, for example, due to different scanners or populations. Second, we acknowledge that FSE-BB sequences are becoming popular for MRI of BMs [10–17], yet we believe until FSE-BB images are fully established, improving widely applicable GE DL methods remains very important. Third, we acknowledge the retrospective nature of our study. In order to validate performance benefits, a prospective study would need to be conducted. Last, we acknowledge that the SPACE sequence is a powerful tool for detecting BMs, but it is not a standalone diagnostic tool for differentiating all types of brain lesions. Accurate characterization of lesions may require additional imaging modalities or techniques that were not evaluated in this study.

To conclude, this study emphasizes that annotation quality plays a crucial role in the development and evaluation of DL methods for BM detection and segmentation. HAQ—created with the help of cross-sequence information of the FSE-BB SPACE sequence—can significantly boost DL method performance on contrast-enhanced T1-weighted MPRAGE imaging, closing the gap between FSE-BB and GE DL methods by a large margin. It allows benefitting from FSE-BB images indirectly, through improved DL methods, without having to invest additional time to acquire such images. This could, if the performance gap in daily practice is as small as in our experiments, make them widely applicable and allow fast and accurate fully automated detection of small BMs.

## Supplementary information

**Additional file 1: Table S1** Internal Detection Performance). Detection performance metrics of model A-D on the internal SPACE and MPRAGE test sets. We report mean ± SD (F1-Score, Positive Predictive Value (PPV) and Sensitivity (SN) of each model on each test dataset. **Fig. S1.** Outline of the selection and preparation process of patient data for deep learning method development utilizing patients from the CYBER-SPACE study. A total of 157 patients were provided, with imaging performed using MPRAGE and SPACE modalities. In the original study patients were randomized into two cohorts to delineate BMs either on MPRAGE or SPACE for later radiation treatment. Of the original study, 76 patients delineated on SPACE and 81 delineated on MPRAGE were received of which 5 were dropped randomly for equal cohort sizes. Due to lower conspicuity of BM on MPRAGE annotations are of normal annotation quality (NAQ), whereas annotations created on SPACE result in high annotation quality (HAQ). Given these images and annotations models were trained in three settings: A) MPRAGE image as input with NAQ; B) MPRAGE image as input with HAQ; and C) SPACE images and HAQ. The flowchart details the distribution of patients at each differentiation step of the study. *MPRAGE* Magnetization-prepared rapid acquisition gradient echo, *SPACE* Sampling perfection with application-optimized contrasts using different flip angle evolution. For the classification model training patches were sampled from MPRAGE & SPACE from the patients with HAQ delineation, involving 440 patches (Foreground: 219, Background: 221) for the training set and 82 patches (Foreground: 42, Background: 40) for the test set. The flowchart details the number of patients at each stage and the specific criteria used for their progression through the study. **Fig. S2.** The box-and-whisker plots illustrate the F1 scores for various convolutional neural network architectures when classifying patches with/without brain metastases. The input sequences for classification tasks are differentiated by color: MPRAGE late contrast in dark blue, MPRAGE with all contrasts in light blue, and SPACE in red. The F1 score served as the performance metric. The SPACE sequence as input demonstrates higher median F1 scores across all architectures, relative to the same architecture trained with MPRAGE images, suggesting better conspicuity of BM on SPACE provides deep learning models a better basis when controlling for annotation quality. **Fig. S3.** Qualitative examples of the predictive behavior of models A, B, and C applied to the internal MPRAGE and SPACE test sets. True positives (TPs), where the models' predictions (PD) coincide with the ground truth (GT); false positives (FPs), where a PD does not align with an annotated brain metastasis (GT); and false negatives (FNs), where PD is absent despite the presence of GT are visualized. The GT is marked in green, while PD is depicted in red.

## Data Availability

The dataset used to train the AI models during the current study is not publicly available due to privacy restrictions but is available from the corresponding author upon reasonable request. Three out of four datasets used for evaluation in the current study are available in TCIA and the stanford.edu repository: 10.7937/6be1-r748, 10.7937/xb6d-py67, https://aimi.stanford.edu/brainmetshare. The fourth dataset used for evaluation in the current study is not publicly available due to privacy restrictions but is available from the corresponding author upon reasonable request.

## Abbreviations

3D: Three-dimensional
BMs: Brain metastases
DL: Deep learning
DSC: Dice similarity coefficient
FSE-BB: Fast spin-echo black-blood
GE: Gradient-echo
GT: Ground truth
MPRAGE: Magnetization-prepared rapid acquisition gradient echo
MRI: Magnetic resonance imaging
PPV: Positive predictive value
SPACE: Sampling perfection with application-optimized contrasts using different flip angle evolution
SUSM: Stanford University School of Medicine
UKHD: University Clinic Heidelberg
UMMC: University of Mississippi Medical Center
YNHH: Yale New Heaven Hospital
