# Supplementary material for: Enhancing deep learning methods for brain metastasis detection through cross-technique annotations on SPACE MRI
Source: Eur Radiol Exp. 2025 Feb 6;9:15. doi: 10.1186/s41747-025-00554-5 (PMC11802942; doi:10.1186/s41747-025-00554-5)
Supplement: Supplementary file 1 — Additional file 1: Table S1 Internal Detection Performance). Detection performance metrics of model A-D on the internal SPACE and MPRAGE test sets. We report mean ± SD (F1-Score, Positive Predictive Value (PPV) and Sensitivity (SN) of each model on each test dataset. Fig. S1. Outline of the selection and preparation process of patient data for deep learning method development utilizing patients from the CYBER-SPACE study. A total of 157 patients were provided, with imaging performed using MPRAGE and SPACE modalities. In the original study patients were randomized into two cohorts to delineate BMs either on MPRAGE or SPACE for later radiation treatment. Of the original study, 76 patients delineated on SPACE and 81 delineated on MPRAGE were received of which 5 were dropped randomly for equal cohort sizes. Due to lower conspicuity of BM on MPRAGE annotations are of normal annotation quality (NAQ), whereas annotations created on SPACE result in high annotation quality (HAQ). Given these images and annotations models were trained in three settings: A) MPRAGE image as input with NAQ; B) MPRAGE image as input with HAQ; and C) SPACE images and HAQ. The flowchart details the distribution of patients at each differentiation step of the study. MPRAGE Magnetization-prepared rapid acquisition gradient echo, SPACE Sampling perfection with application-optimized contrasts using different flip angle evolution. For the classification model training patches were sampled from MPRAGE & SPACE from the patients with HAQ delineation, involving 440 patches (Foreground: 219, Background: 221) for the training set and 82 patches (Foreground: 42, Background: 40) for the test set. The flowchart details the number of patients at each stage and the specific criteria used for their progression through the study. Fig. S2. The box-and-whisker plots illustrate the F1 scores for various convolutional neural network architectures when classifying patches with/without brain metastases. The input sequence [file 41747_2025_554_MOESM1_ESM.pdf]

Enhancing Deep Learning Methods for Brain Metastasis Detection through Cross-Technique Annotations on SPACE MRI

ELECTRONIC SUPPLEMENTARY MATERIAL

Supplement A:

Additional evaluation and statistical analysis details

Across all our experiments we report the final ensemble performance of our 5 different models that were trained via the 5-fold cross-validation, as is standard when developing models with the nnU-Net framework [1].

All values we report are aggregated per-patient level and per-instance, meaning that when two instances are present, we calculate the volumetric metrics for each of the instances first. Given the Dice Similarity Coefficient (DSC) we then assign these instances true positives (TP)/ false positives (FP)/false negative (FN) which is then subsequently used for the calculation of the detection metrics on the per-case level.

Given this per-case aggregation scheme, each model provides a single value for each sample of a metric, which allows us to employ a paired statistical test. In our experiments we use the Wilcoxon Rank-Sum test, which is also known as the Mann-Whitney U test for all our statistical testing.

High Standard Deviation in Results

As evident in our results tables, we notice that our models have high levels of standard deviation (std) across the datasets. This is to be expected given our per-patient aggregation scheme of the Brain Metastasis (BM) instances. Many of the patients only have a low

number of instances, e.g., between 1-3. Whether or not the models predict one of these instances correctly can change the measured performance significantly, in the case of only 1 sample, e.g., the Sensitivity can only be 0 or 1, depending on the models finding the instance. This inherently leads to high amounts of std in the results. Similarly, the volumetric DSC is influenced: If one finds a 2-voxel wide instance with a 1 voxel wide prediction the DSC will immediately be about ~60%. If the prediction is not aligned well DSC will be 0 leading to high amounts of variation as well.

Supplement B:

Classification Experiment

Within our internal test dataset, the *magnetization-prepared rapid acquisition of gradient echo* (MPRAGE) and *Sampling perfection with application- optimized contrasts using different flip angle evolution* (SPACE) images are co-registered to each other. Subsequently smaller registration errors may influence our detection quality especially for smaller instances, where a small registration error may lead to prediction and ground truth to not overlap anymore.

Subsequently we designed another, brief experiment determining the potential upper-bound performance of achievable detection for SPACE and MPRAGE images by building a classifier that is independent of small spatial deviations:

Classification Dataset

Of the cohort of the 76 patients that were annotated with SPACE we create an additional dataset. We crop 261 regions from the images that contain at least one BM instance or more and 261 regions that contain no BM at all, with each axial crop having z-depth 16. We divided these crops into training/validation and test dataset with a 4:1 ratio resulting in 440 training/validation and 82 test samples with both the gadolinium late-phase MPRAGE and the SPACE sequence. For each of the training datasets we trained multiple classification

networks utilizing either, T1ce alone, all MPRAGE images or only the SPACE image for classification.

Classification Networks

The models employed were specifically the 3D versions of ResNet-18, ResNet-50, DenseNet-121, and EfficientNet-B1 of Project Monai (https://monai.io/) [3]. We train a 5-fold cross-validated model with a split of 80:20 for each training dataset and evaluate each fold on the test set individually. Given that each network architecture comprises five folds, this leads to a total of 20 evaluations on the test set for each sequence.

Hyperparameters

During preprocessing, no resampling was necessary as all data was isotropic and consistent in spacing. The dataset was normalized by clipping intensity values to the [0.5, 99.5] percentiles, followed by a z-score normalization on individual images. Batchgenerators (https://github.com/MIC-DKFZ/batchgenerators) [2] was employed for data loading and augmentation. Applied data augmentations included: random rotations (± 0.26 rad in x, y, z) for 10% of samples, random scaling (0.75, 1.25) for 10% of samples, random elastic deformations 0, 0.25 for 10% of samples, brightness transform for 15% of samples, Gaussian noise for 15% of samples, Gaussian blur for 15%$ of samples, gamma transform (0.5, 2) for 15% of samples, mirroring, and 90° rotations on the XY-plane for 30% of samples. In each fold, 20% of samples were designated as validation samples. Models were trained from scratch over 1600 epochs, with a batch size of 2, using binary cross-entropy loss with logits and stochastic gradient descent (learning rate = 2×10^{-4}, Nesterov momentum = 0.9, weight decay = $5 \times 10^{\wedge -4}$) and a cosine annealing schedule starting at epoch 200. No dropout in the last layers since it did not improve results.

Classification Results and Discussion:

The median F1-Score of each architecture tested is lower when using any MPRAGE image configuration as input, than when using the same architectures and with the SPACE images as input. The better F1-Score of the two MPRAGE configurations for DenseNet121 is 0.805 vs 0.842 for the SPACE configuration and for the ResNet50 configuration the better MPRAGE configuration reaches 0.805 F1-Score while the SPACE configuration achieves 0.878 F1-Score. For all explicit median F1-Scores we refer to Figure S2.
From these results we draw the conclusion that the SPACE input imaging sequence is clearly better able to visualize brain tumor instances, while on the MPRAGE sequence it seems to be more difficult to detect tumor instances.

Additionally, we denote that the SPACE performance of the 3D ResNet18 seems to be the best in detecting SPACE by a large margin. We assume that the complexity of identifying BM instances on the SPACE images seems to be very low, hence the smallest architecture provides the best performing configuration. Conversely, the decrease for the same architecture indicates that the task seems to be substantially harder when given MPRAGE images as input, as evident by the steep decrease in performance. Overall, we conclude that our classification models are not optimal yet, hence we only present it as an addendum in case the reader finds it interesting.

Supplement C:

Additional results

In addition to the results provided in the main manuscript, we present quantitative detection results for Model A and Model B across all test datasets. Lesion-wise detection performance is aggregated across all cases in each dataset and reported in Figure S4. It is observable

that Model B consistently exhibits more true positives (TP) and fewer false negatives (FN). Simultaneously, Model B predicts more false positives (FP) than Model A. These results reflect the higher detection sensitivity (SN) already noted in the results of the main manuscript. Furthermore, the high number of FPs could, analogously to the missed instances in our internal dataset, suggest instances where annotations were overlooked during the original annotation process, as well as the general tendency of the model trained on NAQ to predict more conservatively than the model trained on HAQ.

As previously noted, BM lesions are only considered TPs if the DSC (Dice Similarity Coefficient) overlap between the prediction and the ground truth exceeds a minimum value of 0.1.

Supplement D:

Table S1 (Title: Internal Detection Performance): Detection performance metrics of model A-D on the internal SPACE and MPRAGE test sets. We report mean ± SD (F1-Score, Positive Predictive Value (PPV) and Sensitivity (SN) of each model on each test dataset. SD: Standard Deviation.

**Dataset**	**Test Set MPRAGE**			**Test Set SPACE**		
**Metrics**	**Instance F1 Score Mean** (±SD)	**Instance PPV Mean** (±SD)	**Instance SN Mean** (±SD)	**Instance F1 Score Mean** (±SD)	**Instance PPV Mean** (±SD)	**Instance SN Mean** (±SD)
**Model**						
**Setting A**	91.9% ± 11.2%	**97.8**% ± 8.6%	89.0% ± 17.2%	79.8% ± 19.4%	96.4% ± 13.4%	66.7% ± 30.4%
**Setting B**	89.5% ± 14.9%	91.1% ± 18.8%	90.3% ± 15.5%	84.0% ± 17.6%	86.7% ± 19.1%	83.9% ± 19.7%
**Setting C**	87.8% ± 14.6%	84.7% ± 19.5%	**94.0%** ± 12.8%	**91.6%** ± 13.9%	97.8% ± 8.6%	**88.1%** ± 19.3%
**Setting D**	**92.1**% ± 10.5%	92.4% ± 15.3%	**94.0%** ± 10.4%	89.3% ± 12.3%	**100.0%** ± 0.0%	82.6% ± 19.0%

Supplement E:

Annotation Characteristics

Compared to the contrast-enhanced MPRAGE sequence, BM exhibits higher conspicuity on contrast-enhanced SPACE, simplifying the detection of BM lesions for radiologists [4 - 6]. Reichert et al. [7] quantitatively demonstrated that the sensitivity (SN) for detecting BM lesions on MPRAGE ranged from 73.6% to 68.5%, but it significantly improved to between 99.1% and 96.3% with SPACE. Additionally, SPACE's ability to suppress blood vessels minimizes the likelihood of confusing these vessels with BM [8], thereby enhancing diagnostic accuracy. Moreover, the visible tumor volume of BM imaged with SPACE is up to 15.2% larger compared to MPRAGE [8], which facilitates more comprehensive annotations of metastatic volume. As a result, high-quality annotations derived from SPACE not only capture more BM lesions but also feature fewer false negatives and include more metastatic volume than those normally derived from MPRAGE. Furthermore, beyond its application in BM, SPACE is also superior in visualizing glioma volumes compared to MPRAGE [8], underscoring its utility as a versatile tool for brain tumor imaging.

Supplementary References:

[1] Isensee, F., Jaeger, P. F., Kohl, S. A., Petersen, J., and Maier-Hein, K. H. (2021). nnu-net: a self-configuring method for deep learning-based biomedical image segmentation. Nature methods, 18(2):203–211.

[2] Isensee Fabian, Jäger Paul, Wasserthal Jakob, Zimmerer David, Petersen Jens, Kohl Simon, Schock Justus, Klein Andre, Roß Tobias, Wirkert Sebastian, Neher Peter, Dinkelacker Stefan, Köhler Gregor, Maier-Hein Klaus (2020). batchgenerators - a python framework for data augmentation. doi:10.5281/zenodo.3632567

[3] The MONAI Consortium. (2020). Project MONAI. Zenodo. https://doi.org/10.5281/zenodo.4323059

[4] Kraft, J., Zindler, J., Minniti, G., Guckenberger, M., & Andratschke, N. (2019). Stereotactic radiosurgery for multiple brain metastases. Current treatment options in neurology, 21, 1-15.

[5] Komada T., Naganawa S., Ogawa H., Matsushima M., Kubota S., Kawai H., Fukatsu H., Ikeda M, Kawamutra M., Sakurai Y., Maruyama K., Contrast-enhanced MR Imaging of Metastatic Brain Tumor at 3 Tesla: Utility of T1-weighted SPACE Compared with 2D Spin Echo and 3D Gradient Echo Sequence, Magnetic Resonance in Medical Sciences, 2008, Volume 7, Issue 1, Pages 13-21, ISSN 1347-3182, https://doi.org/10.2463/mrms.7.13

[6] Kato Y, Higano S, T. H. (2019). Usefulness of contrast-enhanced t1-weighted sampling perfection with application-optimized contrasts by using different flip angle evolutions in detection of small brain

metastasis at 3t mr imaging: comparison with magnetization-prepared rapid acquisition of gradient echo imaging. AJNR Am J Neuroradiol, 30(5):923–929.

[7] Reichert, M., Morelli, J. N., Runge, V. M., Tao, A., von Ritschl, R., von Ritschl, A., Padua, A., Dix, J. E., Marra, M. J., Schoenberg, S. O., & Attenberger, U. I. (2013). Contrast-enhanced 3-dimensional SPACE versus MP-RAGE for the detection of brain metastases: Considerations with a 32-channel head coil. *Investigative Radiology*, *48*(1), 55–60. https://doi.org/10.1097/RLI.0b013e318277b1aa

[8] L. Danieli, G.C. Riccitelli, D. Distefano, E. Prodi, E. Ventura, A. Cianfoni, A. Kaelin-Lang, M. Reinert, E. Pravatà. Brain Tumor-Enhancement Visualization and Morphometric Assessment: A Comparison of MPRAGE, SPACE, and VIBE MRI Techniques. In American Journal of Neuroradiology Jul 2019, 40 (7) 1140-1148; DOI: 10.3174/ajnr.A6096

Supplementary Figures

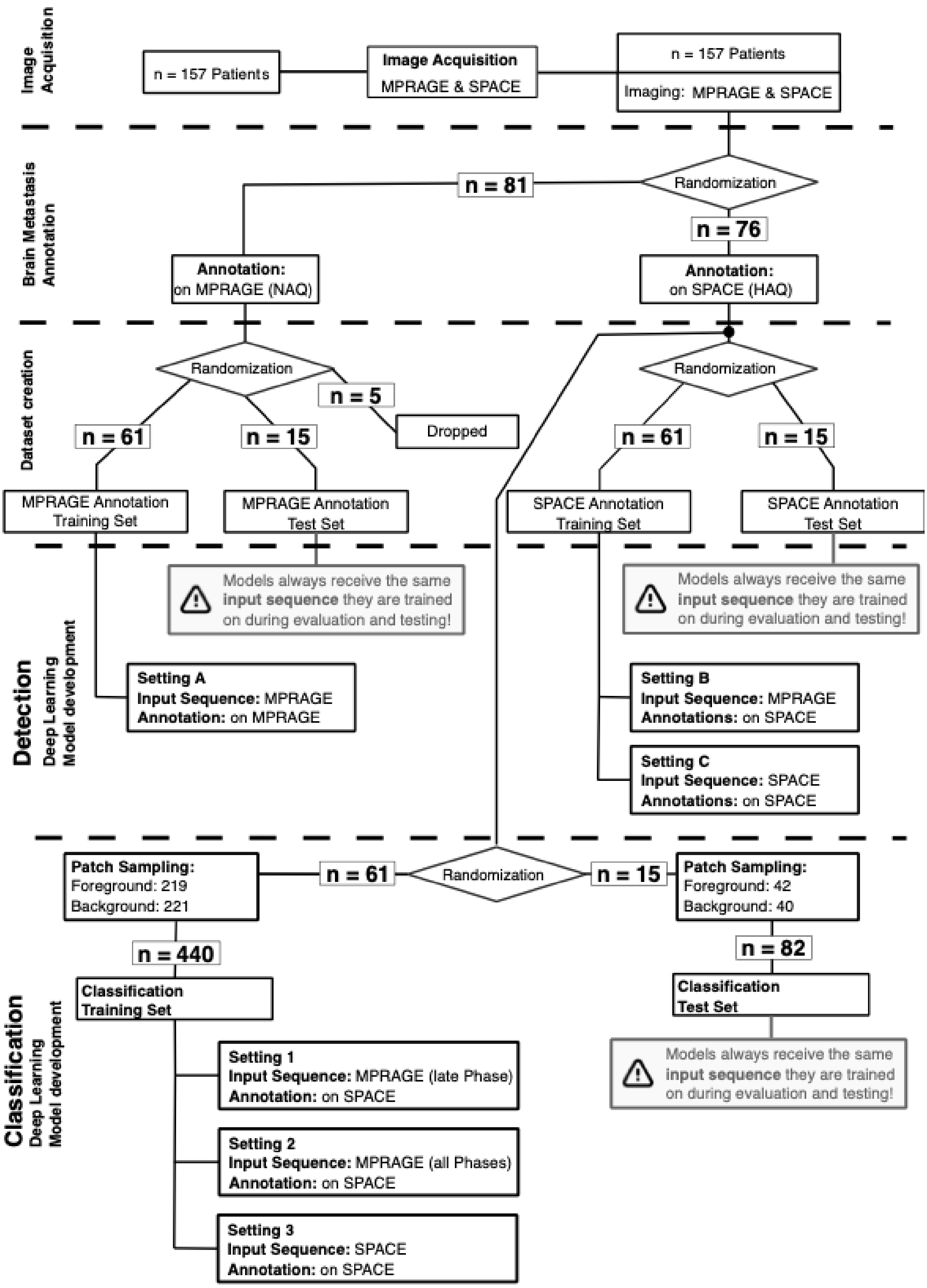

Fig. S1. Outline of the selection and preparation process of patient data for deep learning method development utilizing patients from the CYBER-SPACE study. A total of 157 patients were provided, with imaging performed

using MPRAGE and SPACE modalities. In the original study patients were randomized into two cohorts to delineate BMs either on MPRAGE or SPACE for later radiation treatment. Of the original study, 76 patients delineated on SPACE and 81 delineated on MPRAGE were received of which 5 were dropped randomly for equal cohort sizes. Due to lower conspicuity of BM on MPRAGE annotations are of normal annotation quality (NAQ), whereas annotations created on SPACE result in high annotation quality (HAQ). Given these images and annotations models were trained in three settings: A) MPRAGE image as input with NAQ; B) MPRAGE image as input with HAQ; and C) SPACE images and HAQ. The flowchart details the distribution of patients at each differentiation step of the study. *MPRAGE* Magnetization-prepared rapid acquisition gradient echo, *SPACE* Sampling perfection with application- optimized contrasts using different flip angle evolution. For the classification model training patches were sampled from MPRAGE & SPACE from the patients with HAQ delineation, involving 440 patches (Foreground: 219, Background: 221) for the training set and 82 patches (Foreground: 42, Background: 40) for the test set. The flowchart details the number of patients at each stage and the specific criteria used for their progression through the study.

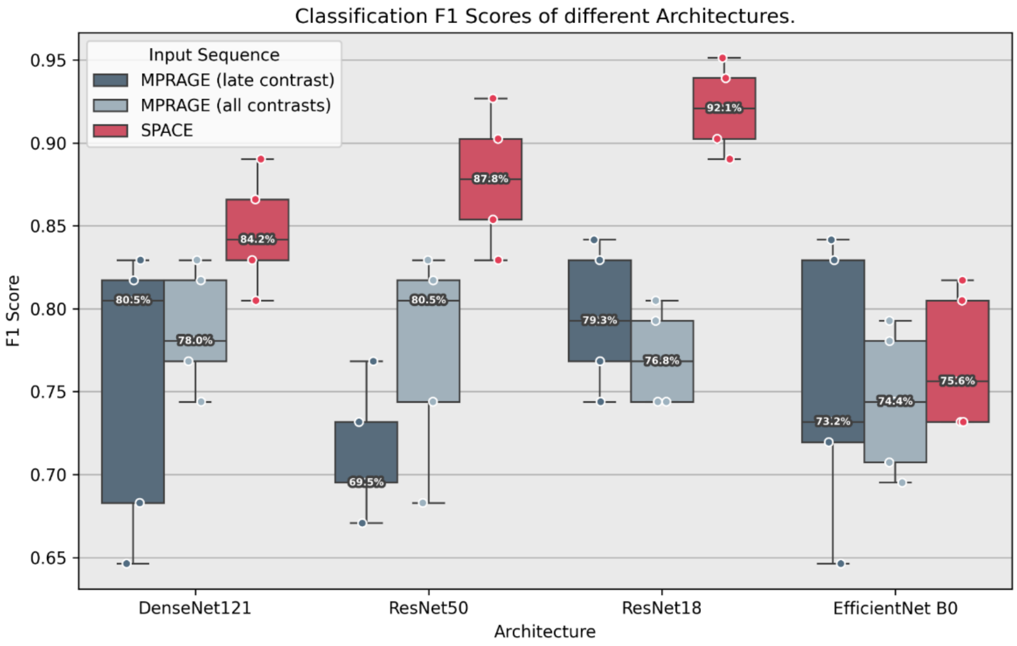

Fig. S2. The box-and-whisker plots illustrate the F1 scores for various convolutional neural network architectures when classifying patches with/without brain metastases. The input sequences for classification tasks are differentiated by color: MPRAGE late contrast in dark blue, MPRAGE with all contrasts in light blue, and SPACE in red. The F1 score served as the performance metric. The SPACE sequence as input demonstrates higher median F1 scores across all architectures, relative to the same architecture trained with MPRAGE images, suggesting better conspicuity of BM on SPACE provides deep learning models a better basis when controlling for annotation quality.

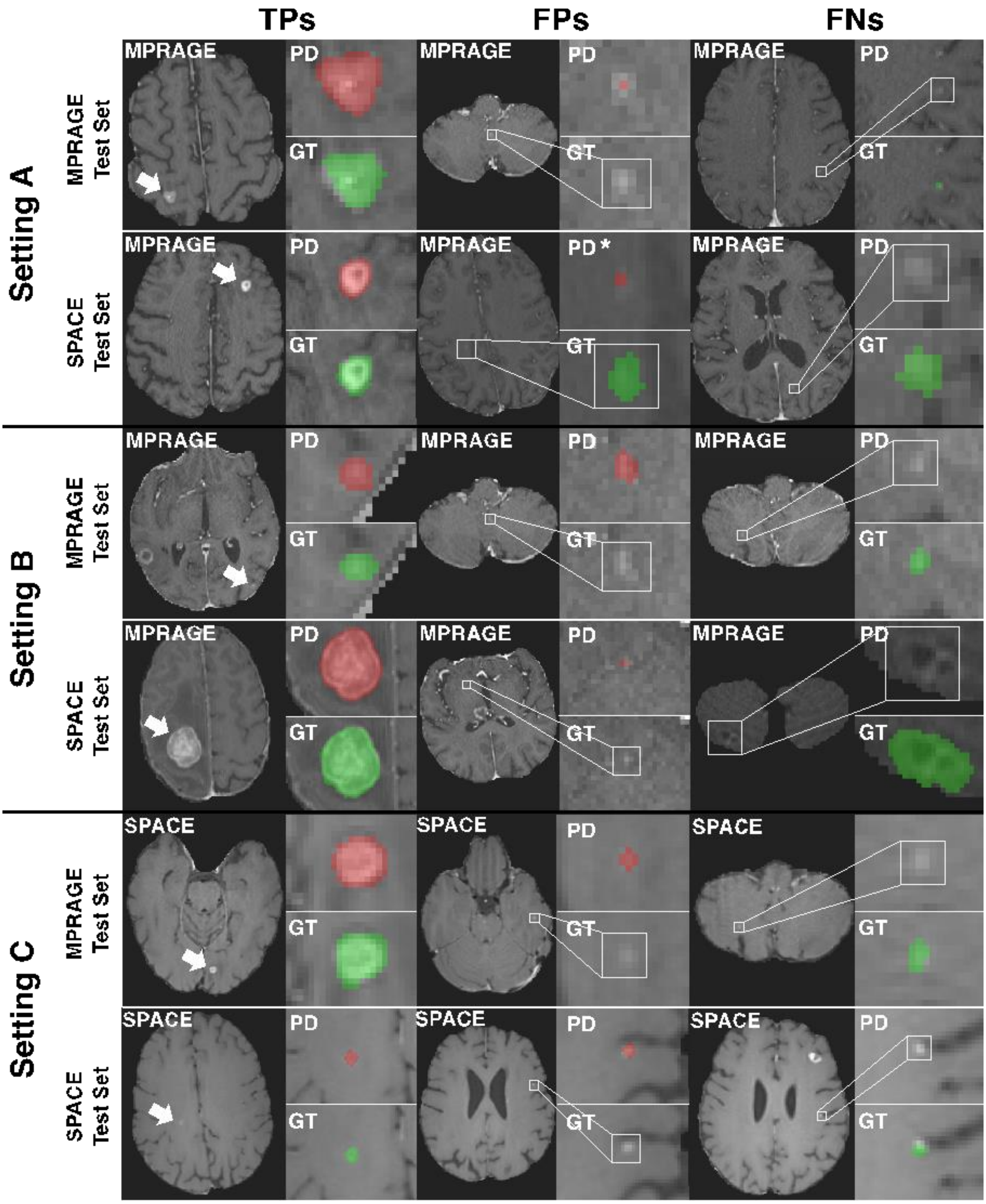

Fig. S3: Qualitative examples of the predictive behavior of models A, B, and C applied to the internal MPRAGE and SPACE test sets. True positives (TPs), where the models' predictions (PD) coincide with the ground truth (GT); false positives (FPs), where a PD does not align with an annotated brain metastasis (GT); and false negatives (FNs), where PD is absent despite the presence of GT are visualized. The GT is marked in green, while PD is depicted in red.

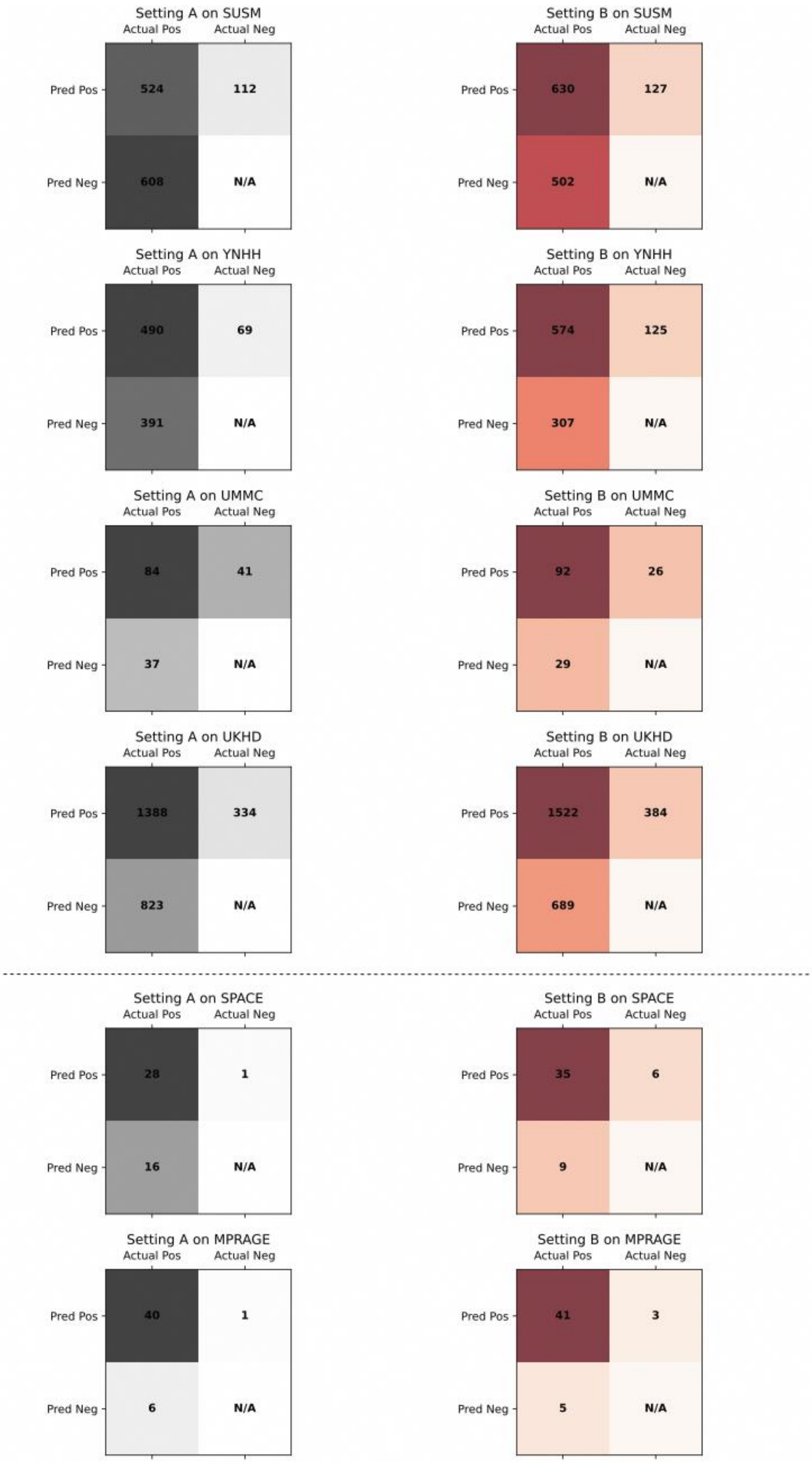

Fig. S4: Confusion matrices displaying lesion-wise detection behavior of Model A and Model B. Values are aggregated across all cases within each dataset. The upper eight matrices are evaluations on external datasets, while the lower four show the results on the internal dataset. It can be observed that the true positive (TP) in the top left of the confusion matrix of setting B are always greater than in setting A. Moreover, the false negatives (FN) in the bottom left are always smaller, indicating the higher detection sensitivity of model B over model A.
